# Development of mental health first aid guidelines on how a member of the public can support a person affected by a traumatic event: a Delphi study

**DOI:** 10.1186/1471-244X-10-49

**Published:** 2010-06-21

**Authors:** Claire M Kelly, Anthony F Jorm, Betty A Kitchener

**Affiliations:** 1 Orygen Youth Health Research Centre, Centre for Youth Mental Health, University of Melbourne, Australia

## Abstract

**Background:**

People who experience traumatic events have an increased risk of developing a range of mental disorders. Appropriate early support from a member of the public, whether a friend, family member, co-worker or volunteer, may help to prevent the onset of a mental disorder or may minimise its severity. However, few people have the knowledge and skills required to assist. Simple guidelines may help members of the public to offer appropriate support when it is needed.

**Methods:**

Guidelines were developed using the Delphi method to reach consensus in a panel of experts. Experts recruited to the panels included 37 professionals writing, planning or working clinically in the trauma area, and 17 consumer or carer advocates who had been affected by traumatic events. As input for the panels to consider, statements about how to assist someone who has experienced a traumatic event were sourced through a systematic search of both professional and lay literature. These statements were used to develop separate questionnaires about possible ways to assist adults and to assist children, and panel members answered either one questionnaire or both, depending on experience and expertise. The guidelines were written using the items most consistently endorsed by the panels across the three Delphi rounds.

**Results:**

There were 180 items relating to helping adults, of which 65 were accepted, and 155 items relating to helping children, of which 71 were accepted. These statements were used to develop the two sets of guidelines appended to this paper.

**Conclusions:**

There are a number of actions which may be useful for members of the public when they encounter someone who has experienced a traumatic event, and it is possible that these actions may help prevent the development of some mental health problems in the future. Positive social support, a strong theme in these guidelines, has some evidence for effectiveness in developing mental health problems in people who have experienced traumatic events, but the degree to which it helps has not yet been adequately demonstrated. An evaluation of the effectiveness of these guidelines would be useful in determining their value. These guidelines may be useful to organisations who wish to develop or revise curricula of mental health first aid and trauma intervention training programs and policies. They may also be useful for members of the public who want immediate information about how to assist someone who has experienced a potentially traumatic event.

## Background

Traumatic events can cause posttraumatic stress disorder and other mental illnesses amongst those who have experienced them, and secondary psychological injury to the friends and family members of the affected. Appropriate early intervention, whether by a friend, family member or co-worker, or by volunteers on-hand when a traumatic event occurs, may help to prevent the onset of a mental disorder or may minimise the severity of the mental disorder, should one develop. However, few people have the knowledge and skills required to assist.

A number of thorough reviews of existing strategies to assist recent victims of trauma exist, including a Cochrane systematic review [1]. Existing psychological interventions intended for use after traumatic events are mainly written for professional helpers. Existing approaches include psychological debriefing (PD), usually conducted as a single debriefing session after the event, and critical incident stress management, which often includes group debriefing. These require substantial training and are only suitable for professional helpers. In addition, they have not been proven to be effective. A number of randomised controlled trials of single-session PD have been conducted, and reviews suggest that they are at best only mildly effective and at worst may cause further harm [1-4]. A small number of RCTs of the use of longer term formalised professional interventions have been conducted [1] and they do appear to be useful. It has also been shown that individuals who meet criteria for acute stress disorder (ASD) or have severe symptoms in the four weeks after a traumatic event are those most at risk of PTSD, and professional intervention for that particular group may help to reduce that risk [4,5].

Despite the lack of success of routine professional debriefing, informal social support appears to be an important factor in altering risk following a traumatic experience, although the research is nascent and further investigation is needed. There is limited evidence that perceived positive social support after a traumatic event may protect against long term psychological injury, while perceived negative social support increases risk [6]. These factors appear to have different mechanisms, and both may operate at the same time; for example, a woman who has been sexually assaulted may perceive positive social support by most, which is helpful, but negative social support in the form of disgust or horror by a few people in her support network. The positive social support by most may be negated by the negative social support she receives from some. What appears to be most important about social support is that it is both perceived as positive, and of the type the individual feels they need [6].

In recent years, guidelines for health professionals on the treatment of ASD and PTSD have been developed in Australia, the UK and the USA [7-9]. There has also been a Delphi expert consensus study of European experts to guide psychosocial care following a disaster [10]. However, these guidelines are not aimed at informing the general public about supportive actions they can take and most of the actions recommended in these guidelines are not appropriate for the public. While a number of guidelines have been written in the past several years for use by incidental helpers, none have been systematically developed or evaluated. These have been written by experts within specific organisations. For example, the Centres for Disease Control (CDC) in the United States publish guidelines for use when a disaster occurs [11]. The National Centre for PTSD, part of the Department of Veteran's Affairs in the United States, has a number of brochures which focus on responding after a traumatic event and supporting individuals with ASD and PTSD [12]. There are a number of others. Sometimes such guidelines are written in response to specific events. The Centre for the Study of Traumatic Stress published guidelines for volunteers deployed in areas affected by the Boxing Day Tsunami of 2004 [13]. Guidelines were also developed in the United States for assisting distressed students and staff in the wake of the Virginia Polytechnic Institute massacre in April 2007 [14], by psychologists at Virginia Tech and by national organisations such as Paper-Clip Communications [15].

In this paper, we aim to improve one particular approach to public education - training of members of the public in how to give first aid to someone who has experienced a traumatic event. One program of this sort is the Mental Health First Aid training program [16], which was developed to train members of the public to provide initial help to a person developing a mental health problem or in a mental health crisis; this help is given until appropriate professional treatment is received or until the crisis resolves. When the program was first in development, the authors used evidence-based information wherever possible, but very little research was found about how members of the public, with no clinical training, could assist a friend, family member or acquaintance who was showing signs of mental disorder or crisis. For advice on how to manage these situations, the authors informally sought the opinions of clinical experts.

## Methods

We chose the Delphi method, a technique used for reaching expert consensus. Our aim was to get consensus within and between panels of professionals, carers and consumers, so that the guidelines would be respectful of the expertise of all three groups. By conducting the research online, it was possible to include participants from English-speaking countries across the world, inexpensively and without lengthy postal delays. The Delphi methodology has been used in health research in the past, mainly to reach consensus amongst medical practitioners, but also with consumers of health services in some settings [17,18]. We have also successfully used this method to develop mental health first aid guidelines for depression, psychosis, suicidal thoughts and behaviours and non-suicidal self-injury using panels of professionals, consumers and carers [19-25].

**Figure 1:**
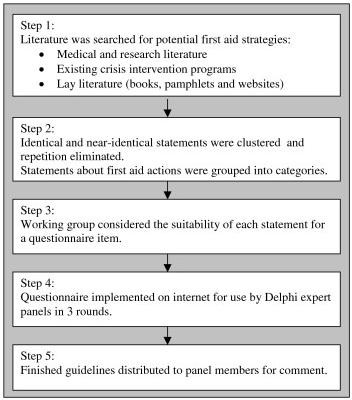
**Stages in guideline development**.

This study had two phases: (1) a literature search for possible first aid actions that the panel could consider and development of a questionnaire covering these actions, and (2) the Delphi process in which the panels reached consensus about the first aid actions likely to be helpful. Please see Figure 1 for a summary of the steps.

### Literature search

The aim of the literature search was to find statements about helping someone who has experienced a traumatic event which would be input for the expert panels to consider. The focus for the search was to find statements which instruct the reader on how to respond immediately after a traumatic event (or the disclosure of a past trauma), how to offer assistance in the short and medium term, and how and when to access professional help for a traumatised individual.

The literature search was conducted across three domains: the medical and research literature, the content of existing crisis intervention guidelines and relevant courses for the public, and lay literature. The lay literature included books written for the general public, particularly consumers' and carers' guides, websites and pamphlets.

The medical and research literature was accessed through searches of PsycInfo and PubMed. This was not a systematic review. No judgment was made about the quality of the evidence or the methods. Any claim about an action that might be effective when assisting someone who has experienced a traumatic event was considered for inclusion in the list of items to be assessed by the panel members (for further details see "Questionnaire development" below).

The search term was 'trauma*' and all records for the 20 years leading to the search date were reviewed. The search term 'trauma*' generated far too many records, including large numbers of records relevant only to physical trauma, but all attempts to narrow the search were found to exclude too many possibly relevant records. Papers were therefore excluded first on the basis of their titles and then on the basis of their abstracts.

Papers were read if they described actions to prevent to development of PTSD after a traumatic event, described risk and protective factors that were modifiable post-trauma (e.g. social bonds and social isolation can be acted on and enhanced after someone has experienced a traumatic event; whereas pre-event trait anxiety cannot), or included guidelines for treating patients who had recently been exposed to trauma (a total of 194 papers). Statements meeting our criteria were drawn from 32 of the 194 relevant records, as most of the advice given in these papers was very clinically orientated, or required extensive training, to be applicable.

To find appropriate websites, we used the search engines Google [26], Google Australia [27], and Google UK [28] using the search term 'traumatic event'; the first 50 websites listed by each were reviewed; beyond the first 50 websites, quality declined rapidly. Since most websites were listed by more than one search engine, only 63 websites were reviewed. The websites were read thoroughly, once again looking for statements which suggested a potential first aid action (what the first aider should do) or relevant awareness statement (what the first aider should know). Any external links to other websites were followed and the same process applied to each of them.

It emerged that there was a great deal of information about how to assist children who had been affected by traumatic events. It was therefore decided that an additional search of websites should be conducted to find statements about helping children. The process was repeated, using the search terms 'traumatic event' and 'children'. This time, 55 websites were identified by the three Google search engines, of which 45 had not appeared in the original search.

The fifty most popular books on the Amazon [29] website which listed the word 'trauma' or 'posttraumatic stress disorder' in the title or keywords were selected. This site was chosen because of its extensive coverage of books in and out of print, including works about mental health aimed at the public. Books which were autobiographical in nature, self-help workbooks constituting a program of self-treatment and clinical manuals were excluded. The remaining books were read to find useful statements. The majority of these were carers' guides, which do contain advice relevant for first aid, but focussed on general caring for a mentally ill family member.

Any relevant pamphlets were sought and read, and statements were taken from these as well. The majority of the pamphlets were written and distributed by organisations focussing on specific sorts of traumas, such as sexual assault or violent crime, and generally directed the reader to appropriate authorities and support organisations. There were also a large number of pamphlets and fact sheets focussing on specific large-scale traumas, which were frequently written in response to a specific event, such as Hurricane Katrina in 2005, and the shootings at Virginia Polytechnic Institute in 2007. While these documents did contain a lot of specific advice about where to get practical or emotional help after such an event, there was also information relevant to first aid givers about how to support people affected by such events. Most of these pamphlets were obtained from websites, but where these were not available online, a request was made for relevant materials from large mental health and community organisations.

Guidelines written for professionals responding to traumatic events were reviewed and relevant statements were drawn from these. While a small number of relevant statements were found in these documents, they frequently emphasised the policies and procedures relevant to the specific organisation for which they were developed.

Only one training course for members of the public was found to be relevant, as most training in critical incident response is designed for professional responders such as paramedics and the police. Material from the Mental Health First Aid Program [30] was reviewed and statements drawn from it.

### Questionnaire development

The questionnaire on possible first aid actions was developed by first grouping statements into categories: immediate assistance after a traumatic event; communicating with a traumatised person; discussing the traumatic event; assisting after a large-scale traumatic event; after-care for large-scale traumatic events; coping strategies in the weeks following the event (talking and actions); and when to seek professional help.

The categories for the children's statements were slightly different, and included: immediate assistance after a traumatic event; communicating with a traumatised child; children at large-scale traumatic events; advice for parents and guardians in the weeks following the event; dealing with avoidance behaviour and temper tantrums; legal issues if a child discloses abuse; and when to seek professional help for a child.

Similar or near-identical statements were frequently derived from multiple sources, and they were not repeated in the questionnaire. A working group comprised of the authors of this paper and colleagues working on similar projects convened at each stage of the process to discuss each item in the questionnaire. The role of the working group was to ensure that the questionnaire did not include ambiguity, repetition, items containing more than one idea or other problems which might impede comprehension. The working group made no judgements about the value of the first aid actions in the statements, since that was the role of the expert panels.

The wording of each item was carefully designed to be as clear, unambiguous and action-oriented as possible. For example, 'the first aider should talk about what happened' is highly ambiguous. It is better to specify 'the first aider should encourage the person to talk about the traumatic event', or 'the first aider should tell the person that if they want to talk about the event, the first aider is prepared to listen'. All statements were written as an instruction as shown in the above examples. The only items which were not included in the questionnaire were those which were so ambiguous that the working party was not able to agree on the meaning of the statement, those which were deemed too clinical or relevant only to a specific professional group, and those which called upon 'intuition', 'instinct' or 'common sense', as these cannot be taught.

All participants answered the questionnaire via the Internet, using an online survey website, Surveymonkey [31]. Participants were able to stop filling in their questionnaires at any time and log back in to continue, without the risk of losing the completed section of their questionnaire. Using the Internet also made it very easy for the researchers to identify those who were late in completing questionnaires and send reminders, with no need to send extra copies of the questionnaire. No questions were inadvertently missed, as the web survey was set up so that each question was mandatory. In addition, such survey software allows for branching, so participants who did not feel qualified to answer questions about assisting children who had experienced trauma were not asked to complete those sections of the questionnaire.

### Expert panel recruitment

Participants were recruited into one of three panels: professionals (clinicians and researchers), consumers (people who had experienced a traumatic event, some of whom had post-traumatic stress disorder) and carers (family members or loved ones of consumers who have a primary role in maintaining their wellbeing). Consumers and carers had public roles, either in advocacy, as the authors of books or websites or as speakers on the topic. The professional panel had 37 experts, the consumer panel 13, and the carer panel 4. The carers were also consumers themselves, and because of the small numbers, the consumer and carer panels were combined into one panel of 17.

All panel members were from developed English speaking countries (Australia, Canada, New Zealand, The United Kingdom and The United States). Only participants from developed English speaking countries were sought, as these countries were known to have comparable cultures and health systems. It was also felt that a guaranteed degree of fluency was important because some items vary from each other in important, but very subtle ways, which might escape the notice of a non-native speaker.

Participants were recruited in a number of ways. Professionals recruited were those who had publications in the areas of traumatic stress, PTSD, or treatment of patients who had experienced traumatic events. When letters were sent (by email) to professionals asking them to be involved, they were also invited to nominate any colleagues who they felt would be appropriate panel members. Those active in clinical practice were also asked to consider any former patients who might be willing to be involved and also met our other criteria.

No attempt was made to make panels representative. The Delphi method does not require representative sampling; it requires panel members who are information- and experience-rich. This may be one reason that consumers and carers were difficult to recruit. To be included on the panel, they needed experience beyond their own; for example, involvement in facilitating mutual help support groups or advocacy roles.

It is not possible to report accurately the rate of acceptance or rate of refusal, as it is not known how many of the invitations were received. Changes and errors in email addresses, email filtering programs and other factors make it impossible to report how many of the invitations were read by the person they were addressed to. However, we can report that 190 email invitations were initially sent out. Some of those approached may have passed the information on to others. Some approaches were made to organisations, and may or may not have been read by the relevant individuals. Reasons for refusal included being too busy (this project represented a significant time commitment), no longer working in the area, or working in a related area of less relevance to the project (e.g. brain imaging studies). As the research was to be conducted online, only email contact was initiated.

The 37 professional participants included 21 academics (researchers, lecturers and professors), 15 psychologists, 8 psychiatrists, 7 managers of mental health services or clinical research centres, 2 social workers, 2 nurses, 2 public health policy and program professionals in disaster planning, 1 drug and alcohol therapist working with victims of trauma who abuse drugs, and 1 attorney (also a clinical psychologist). Some participants had multiple roles in research, teaching and clinical work.

Consumers were recruited from advocacy organisations and referral by clinicians. They were also identified if they had written websites offering support and information to other consumers. Carers were recruited through carers' organisations, but were difficult to recruit for this study.

### The Delphi process

Three rounds of questionnaires were distributed as follows, with each item being rated up to two times. In round 1 the questionnaire, derived from the process described above, was given to the panel members. The questionnaire included space after each of the sections to add any suggestions for additional items.

In each round of the study, the usefulness of each item for inclusion in the mental health first aid guidelines was rated as *essential*, *important*, *don't know *or *depends*, *unimportant*, or *should not be included*. The options *don't know *and *depends *were collapsed into one point on the scale because operationally, they are the same response; most of the items were, very reasonably, noted to be useful in some cases and not others, meaning they could not be generalised in guidelines, which is also true of items participants did not feel confident to rate.

The suggestions made by the panel members in the first round were reviewed by the working group and used to construct new items for the second round. Suggestions were accepted and added to round 2 if they represented a truly new idea, could be interpreted unambiguously by the working group, and were actions. Suggestions were rejected if they were near-duplicates of items in the questionnaire, if they were too specific (for example, "Should make sure that the child will be picked up from school"), too general ("just be there"), or were more appropriate to therapy than first aid ("reframe memories of trauma into life lessons, get to the real root of anger, fear, create learnings from experience").

Items rated as *essential *or *important *by 80% or more of the professional and consumer/carer panels were considered to have met consensus for inclusion in the guidelines. If they were endorsed by 80% or more of one of the panels, or by 70-80% of both panels, they were re-rated in the subsequent round. Items which met neither condition were considered to have met consensus for rejection from the guidelines and were not re-rated because previous research by our group has shown that major changes in ratings do not occur in the next round. Before the second and third rounds of the study, each participant was sent a summary of the results of the previous round, listing which items had been accepted, which had been rejected, and which were to be re-rated. It is important to note that only items that approached consensus for the criterion for inclusion were submitted for re-rating by the panels. When an item was to be re-rated by the panellists, they were provided with their own response and a table outlining how many people in each group had endorsed the item. They were told that they did not have to change their responses when re-rating an item, but that if they wished to, they would have the opportunity to do so.

## Results

Tables 1 and 2 show the continuity of participation across the three rounds. Note that some panel members answered only the questions relevant for helping children, some answered only the questions relevant for helping adults, and some completed both. The attrition rate for both studies was significant. Non-responders were contacted to remind them to complete the survey up to three times. Some attrition was due to changes in email addresses, some people found themselves too busy to continue to participate and others did not respond to enquiries.

**Table 1:** Study participation in each round, adult guidelines

Panel	Round 1	Round 2	Round 3
Consumers and carers)*	17 (4)	15 (4)	12 (4)
	100%	82%	71%
Professionals	39	27	23
	100%	69%	59%

* 4 carers who were also consumers are included in this group. Their participation in each round is indicated by the bracketed figure, i.e. in round one this group included 17 consumer participants, of whom 4 were also carers.

**Table 2:** Study participation in each round, child guidelines

Panel	Round 1	Round 2	Round 3
Consumers and carers*	12 (4)	12 (4)	10 (4)
	100%	100%	83%
Professionals	22	22	17
	100%	100%	77%

* 4 carers who were also consumers are included in this group. Their participation in each round is indicated by the bracketed figure, i.e. in round one this group included 12 consumer participants, of whom 4 were also carers.

**Figure 2:**
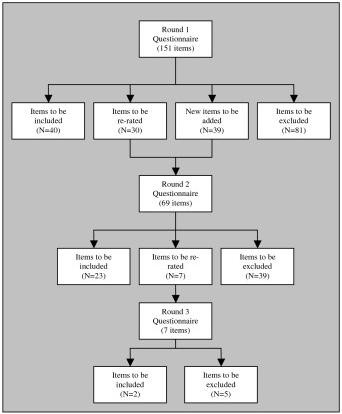
**Items accepted, rejected and re-rated at each round (adult questionnaire)**.

**Figure 3:**
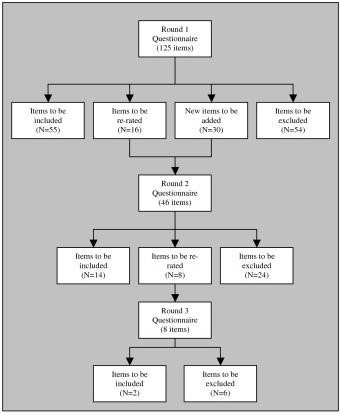
**Items accepted, rejected and re-rated at each round (child questionnaire)**.

Figure 2 shows the rates of inclusion, exclusion and re-rating of the items in each round of the adult questionnaire, while Figure 3 shows the rates of inclusion, exclusion, and re-rating of the items in each round of the child questionnaire. See Tables 3 and 4 for a categorised list of accepted items for the adult and child guidelines.

**Table 3:** Statements accepted as mental health first aid guidelines for assisting adults

**Item:**	**Round:**
**Actions to be taken immediately**	
The first aider should determine whether or not it is safe to approach the person before taking any action (for example, danger from fire, weapons or debris)	1
The first aider should explain to the person what their role is and why they are present.	1
The first aider should create a safe environment.	1
The first aider should be calm in the face of the trauma.	1
The first aider should ascertain the person's basic human needs for the immediate future and attempt to meet them.	2
If helping someone they do not know, the first aider should find out the person's name and use it when talking to them.	2
The first aider should attempt to ascertain and meet the basic human needs of the person (for food, drink, shelter and clothing), but should not take over the role of professionals helpers better able to meet those needs.	2
If the person has been a victim of crime, the first aider should consider the possibility that forensic evidence may need to be collected (for example, cheek swabs, evidence on clothing or skin) and should work with the person in preserving such evidence.	3
The first aider should watch for signs that the person's physical or mental state is declining, and be prepared to seek emergency medical assistance for them (for example, an apparently uninjured person may have internal injuries which reveal themselves more slowly, or a person may suddenly become disoriented).	2
**Guidelines for communicating with the traumatised person**	
The first aider should speak clearly and avoid clinical and technical language.	1
The first aider should communicate with the person as an equal, rather than as a superior expert.	1
The first aider should remember that behaviour such as withdrawal, irritability and bad temper may be a response to the trauma, and should avoid taking such behaviour personally.	1
The first aider should be friendly, even if the person is being difficult.	2
The first aider should show that they understand and care.	1
The first aider should be aware that the person may not be as distressed about the trauma as might be expected.	1
The first aider should remember that they are not the person's therapist.	2
The first aider should tell the person that everyone has their own pace for dealing with trauma.	1
The first aider should encourage the person to talk about their reactions only if the person feels ready to do so.	2
The first aider should remember that providing support doesn't have to be complicated, and can involve small things like spending time together, having a cup of tea or coffee, chatting about day-to-day life or giving them a hug.	1
The first aider should remember that it is more important to be genuinely caring than to say all the "right things".	2
The first aider should be aware of cultural differences in the way some people respond to a traumatic event; for example, in some cultures, expressing vulnerability or grief around strangers is not considered appropriate.	2
The first aider should be prepared to repeat themselves several times if the person seems unable to understand what is said.	3
The first aider should ask the person how they would like to be helped.	2
**Talking about the trauma**	
The first aider should not force the person to tell their story.	1
The first aider should not interrupt to share their own feelings and opinions.	2
The first aider should be aware that the person may need to talk repetitively about the trauma and be willing to listen.	1
The first aider should avoid saying things which minimise the person's feelings, such as "don't cry" or "calm down".	1
The first aider should avoid saying things which minimise the person's experience, such as "you should just be glad you're alive."	1
The first aider should not tell the person how they should be feeling.	1
The first aider should be aware that the person may be experiencing survivors' guilt.	1
The first aider should not make promises they can't keep such as "I'll take you home soon".	1
**Immediate assistance at large scale traumatic events**	
The first aider should follow the directions of professional helpers at the scene.	1
The first aider should get medical help for the person if this is needed.	1
The first aider should find out what emergency help is available.	1
The first aider should provide truthful information and admit that they lack information if this is the case.	1
The first aider should identify basic needs (food, drink, shelter and clothing) and attempt to meet them.	1
The first aider should be aware of and responsive to the person's comfort and dignity, e.g., by offering the person something to cover themselves with (such as a blanket) and asking bystanders and the media to go away.	1
If the person does not want more information about the event, the first aider should not try to give them any.	2
The first aider should tell the person about any available sources of information which are offered to survivors (for example, information sessions, fact sheets and phone numbers for information lines).	2
The first aider should try not to appear rushed or impatient.	2
**After trauma care at a large scale event**	
No items accepted.	
**Coping strategies: talking**	
The first aider should encourage the person to identify sources of support including loved ones and friends.	1
The first aider should respect the person's need to be alone at times.	1
The first aider should encourage the person to tell others when they need or want something, rather than assume others will know what they want.	1
**Coping strategies: actions**	
The first aider should encourage the person to think about what coping strategies they have successfully used in the past and encourage them to continue to use these.	1
The first aider should encourage the person to do whatever they need to do to take care of themselves.	2
The first aider should encourage the person to do things that feel good to them (for example, take baths, read, exercise, watch television).	1
The first aider should encourage the person to get plenty of rest when they are tired.	1
The first aider should encourage the person to spend time somewhere they feel safe and comfortable.	1
The first aider should discourage the person from using negative coping strategies such as working too hard, using alcohol and other drugs, or engaging in self-destructive behaviour.	1
The first aider should assist the person to find local sources of support.	2
The first aider should give the person information about community resources that are available (for example, crisis lines and health centres).	2
The first aider should be aware that the person may not remember all the details of the event.	2
The first aider should be aware that the person may suddenly or unexpectedly remember details of the event.	2
**When to seek professional help**	
If at any time the person becomes suicidal, the first aider should seek professional help.	1
The first aider should encourage the person to seek professional help if the post-trauma symptoms are interfering with their usual activities for 4 weeks or more.	1
The first aider should encourage the person to seek professional help if they feel very upset or fearful for 4 weeks or more.	1
The first aider should encourage the person to seek professional help if they are unable to escape intense ongoing distressing feelings for 4 weeks or more.	1
The first aider should encourage the person to seek professional help if their important relationships are suffering as a result of the trauma (eg, if they withdraw from their carers or friends) for 4 weeks or more.	1
The first aider should encourage the person to seek professional help if they abuse alcohol or other drugs to deal with the trauma at any time.	2
The first aider should encourage the person to seek professional help if they feel jumpy or have nightmares because of or about the trauma for 4 weeks or more.	1
The first aider should encourage the person to seek professional help if they can't stop thinking about the trauma for 4 weeks or more.	1
The first aider should encourage the person to seek professional help if they are unable to enjoy life at all as a result of the trauma for 4 weeks or more.	2
The first aider should be aware of the sorts of professional help which are available.	2
If the person does not like the first professional they speak to, the first aider should tell the person that it is okay to try a different one.	2

**Table 4:** Statements accepted as mental health first aid guidelines for assisting children

**Item:**	**Round:**
**Assisting the traumatised child**	
The first aider should protect the child from further harm.	1
The first aider should ensure the child's physical needs (food, drink and somewhere to sleep) are met.	1
The first aider should not make judgments about the child's feelings and thoughts.	1
The first aider should tell the child that it is okay to feel upset when something bad or scary happens.	1
The first aider should not say that someone who has died has "gone to sleep" as this may result in the child becoming fearful of sleep.	1
The first aider should not make promises to the child that they cannot keep.	1
The first aider should ensure that that they or another adult are available to take care of the child.	1
The first aider should tell the child that they or another adult will take care of them.	2
**Children at large-scale traumatic events**	
The first aider should attempt to keep the child together with loved ones and carers.	1
The first aider should protect the child from traumatic sights and sounds (including media images).	1
The first aider should ask bystanders and the media to stay away from the child.	1
The first aider should not behave towards the child in such a way that the child feels they are still in danger.	1
The first aider should reassure the child that they won't be left alone, so far as this is possible.	1
If the first aider has to leave the child alone for a few minutes to attend to others, they should reassure the child that they will back as soon as possible.	1
The first aider should try to appear as calm as possible.	2
The first aider should direct the child away from very distressed people (e.g., people who are screaming, agitated or aggressive).	2
The first aider should ask the child what would make them feel better or safer.	2
**Communicating with the traumatised child**	
The first aider should talk to the child using age-appropriate language and explanations.	1
The first aider should not coerce the child to talk about their feelings or memories of the trauma before they want to do so.	1
The first aider should be aware that child may stop talking altogether after a trauma, and that if this happens they should not try to force or coerce the child to speak.	1
The first aider should allow the child to ask questions and should answer them as truthfully as possible.	1
The first aider should not make the child discuss the trauma before they are ready.	1
The first aider should say that they can't answer a child's question if this is the case.	1
If the child knows accurate, upsetting details, don't deny these.	1
The first aider should be patient if the child asks the same question many times.	1
The first aider should try to be consistent with answers and information.	1
The first aider should allow the child to talk about their feelings.	1
The first aider should allow the child to write or draw pictures about their feelings.	1
The first aider should allow the child to express their feelings through playing with toys.	1
The first aider should not tell the child how they should or shouldn't be feeling.	1
The first aider should not tell the child to be brave or tough or not to cry.	1
The first aider should not get angry if the child expresses strong emotions.	1
The first aider should show the child that they understand and care.	2
The first aider should tell the child that they will do their best to keep the child safe.	2
The first aider should be patient with the child.	2
The first aider should encourage the child to do things they enjoy (for example, playing with toys, reading books).	2
**If the first aider lives with the traumatised child**	
The first aider should try to keep their behaviour as predictable as possible.	1
The first aider should encourage the child to keep to daily routines.	2
The first aider should not get angry, critical, or call the child 'babyish' if the child begins bedwetting, misbehaving, or sucking their thumb.	1
The first aider should help the child to feel in control by letting them make some decisions (e.g. about meals or what to wear).	1
The first aider should tell the child that their loved ones and carers love and support them.	1
**Dealing with avoidance behaviours and tantrums**	
The first aider should be aware that the child may avoid things that remind them of the trauma (such as specific places, driving in the car, certain people, or separation from their carers.	1
The first aider should try to discover what triggers sudden fearfulness or regression in the child.	1
If the child avoids things which remind them of the trauma, but does not appear very distressed, the first aider should assure them that they are safe.	1
If the child has a temper tantrum or becomes fearful, crying and clingy in order to avoid something which reminds them of the trauma the first aider should ask what they are afraid of.	1
**Legal issues relating to child abuse**	
The first aider should know the local laws or regulations about reporting suspected child abuse.	1
If the child discloses abuse, the first aider should contact the appropriate authorities.	1
If the child discloses abuse, the first aider should remain calm and reassure the child that they have done the right thing by telling.	1
If the child discloses abuse, the first aider should seek expert advice immediately.	1
If the child discloses abuse, the first aider should not confront the perpetrator.	1
If a child has disclosed abuse, the first aider should work with the appropriate authorities to ensure the child's safety.	2
If a child has disclosed abuse, the first aider should assure the child that the abuse was not their fault.	2
If a child has disclosed abuse, the first aider should tell the child that they believe what the child has told them.	3
**Getting professional help for a traumatised child**	
If at any time the child becomes suicidal, the first aider should seek professional help.	1
The first aider should seek professional help for the child if they display sudden severe or delayed reactions to trauma for 2 weeks or more.	1
The first aider should seek professional help for the child if the post-trauma symptoms are interfering with their usual activities for 2 weeks or more.	2
The first aider should seek professional help for the child if they are unable to escape intense ongoing distressing feelings for 2 weeks or more.	1
The first aider should seek professional help for the child if their important relationships are suffering as a result of the trauma (eg, if they withdraw from their carers or friends) for 2 weeks or more.	1
The first aider should seek professional help for the child if they are unable to enjoy life at all as a result of the trauma for 2 weeks or more.	1
The first aider should seek professional help for the child if they feel very upset or fearful for 4 weeks or more.	1
The first aider should seek professional help for the child if they act very differently after the trauma for 4 weeks or more.	1
The first aider should seek professional help for the child if they feel jumpy or have nightmares because of or about the trauma for 4 weeks or more.	1
The first aider should seek professional help for the child if they can't stop thinking about the trauma for 4 weeks or more.	1
The first aider should seek professional help for the child if has temper tantrums or becomes fearful, crying and clingy in order to avoid something which reminds them of the trauma for 4 weeks or more.	1
If the first aider is not a parent or guardian, they should not seek professional help for the child independently of the parent or guardian, except in an emergency.	2
The first aider should be aware of the types of professional help which are available for children.	3
The first aider should be aware that the symptoms associated with trauma may suddenly or unexpectedly appear months or years after the event and that if this occurs, professional help may need to be sought.	2

### Writing the Guidelines

It was important to the research team to avoid making the guidelines read like a list of 'dos' and 'don'ts'; the large number of items would have made the document very cumbersome, and a narrative approach improved readability. The accepted items were incorporated into a plain language document. To illustrate, consider the following statements:

1. The first aider should avoid saying things which minimise the person's feelings, such as "don't cry" or "calm down".

2. The first aider should avoid saying things which minimise the person's experience, such as "you should just be glad you're alive."

3. The first aider should not tell the person how they should be feeling.

These statements were incorporated to make the following paragraph:

Avoid saying anything that might trivialise the person's feelings, such as "don't cry" or "calm down", or anything that might trivialise their experience, such as "you should just be glad you're alive."

When the guidelines were in draft form, they were sent to all the panel members for feedback. Only feedback related to readability and structure was sought and incorporated, and these amounted to only minor typographical changes. The guidelines are appended to this article and can be freely distributed (see additional files 1 and 2).

The guidelines as a whole contain three major sections. The first section includes actions that should be taken immediately after an event has occurred, particularly where there has been threatened or actual loss of life or injury. They follow a simple pattern: ensure your own safety first, look after any physical injuries, get emergency assistance if it is not there already, and be clear and calm in your communications. The second section is about assisting in the weeks following the traumatic event. This section includes advice about positive coping strategies (such as encouraging the person to use existing support networks and community resources and avoiding the use of negative coping strategies such as alcohol and other drugs). These two domains of first aid are appropriate to provide for anyone who has experienced a traumatic event. Most people will recover normally from a traumatic event after a given period of time. The third section differentiates between people who are recovering normally and those who are in need of professional assistance, and includes advice about the signs that professional help may be needed (such as intrusive thoughts, difficulty sleeping and nightmares for four weeks or more after the event).

The advice amounts to providing positive social support to those who have experienced a traumatic event and referral to professional helpers for those with lingering symptoms, both of which reflect what is currently known about decreasing the risk of long term mental health consequences of traumatic events.

## Discussion

This is the first Delphi expert consensus study to examine how members of the public should best respond to someone affected by a traumatic event. While there have been no previous studies primarily aimed at the public's response, there have been previous expert consensus guidelines aimed at professionals. Comparing the present findings to those of the Delphi study to develop European guidelines on post-disaster psychosocial care [10], the main overlap is that both endorse the value of social support. However, the current guidelines suggested much more specific actions that a member of the public could carry out to give social support. The present guidelines can also be compared to the Australian professional guidelines, which included some advice for consumers and carers based on the views of an expert committee [9]. The main elements of advice to carers were to listen and show care, encourage professional help-seeking and stay focussed on recovery, and carer self-care. The main difference from the present guidelines is the advice to consumers to get professional help if they do not get better after 2 weeks, in contrast to the 4 weeks recommended here for adults and 2 weeks for children.

In previous Delphi studies to develop mental health first aid guidelines for the public, we have found some differences in ratings between panels [19-25]. However, the differences in ratings between the two panels in the present study was not as dramatic as they have been in earlier studies [19-25]. Few items were rejected on the basis of a rejection by only one panel. However, a number of items that did not reach the 80% endorsement rate in either panel had significantly differing rates of endorsement. For example, consumers and carers were more likely to endorse actions which would have first aiders encouraging people (including children) to talk about what happened, to express their emotions, and to validate those emotions. It may be that professionals recognised that such encouragement might turn a conversation into an amateur debriefing session, which could be dangerous for all involved. Items were endorsed, however, which instructed first aiders to allow the person to talk if they want to.

The specific content of the guidelines for assisting children is somewhat different, but the overall structure is very similar. In the opening statement, it is stated that if the mental health first aid is being provided by a parent, the parental role takes precedence over the first aid role. While some of the advice may be useful to parents who are finding it difficult to cope and wish for some guidance, generally the guidelines are more appropriate to other caregivers, such as incidental helpers at the scene of a traumatic event, teachers, and other adults in the child's life.

One major difference between the adult and child guidelines is about when to seek professional help. The adult guidelines suggested that many post-traumatic symptoms such as nightmares, feeling jumpy, and being unable to stop thinking about the event should lead the first aid giver to recommend professional help if they persisted for four weeks or longer. By contrast, in the guidelines for children, professional help was recommended if the symptoms persisted for two weeks.

The effectiveness of mental health first aid provided by members of the public after a traumatic event is as yet unproven. However, the common-sense advice about assisting in practical ways immediately after a trauma, and the social support recommended for the following weeks, and advice about professional assistance advocated by the guidelines are sensible, practical and in line with existing evidence. Future research will be needed to determine whether the guidelines are effective in minimising the psychological sequelae of experiencing a traumatic event.

### Limitations

There are a number of limitations in membership of the panels. The panels were not sampled from a defined population list, so the response rate and representativeness are impossible to determine. Furthermore, the size of the panels was small, particularly the carers' panel. It may be that some carers were not present at the time of the traumatic event, or may not feel that they have any expertise, since the event was an isolated incident. Some of the consumers reported that their loved ones were not aware of the traumatic event they had experienced, only of the resulting mental health problems. The professionals' panel was not large, either, but it was very diverse, including respondents with a broad range of professional backgrounds, training, and country of origin.

There was significant attrition after Round 1. Most of the accepted items (40 out of 65, or 62% in the adult study and 55 out of 71, or 77% in the child study) were endorsed in the first round. The attrition rate may have influenced the outcome of some of the marginal items in rounds 2 and 3.

In addition, it is important to note that consensus does not mean validity. Although the feedback from the panel members indicated that the guidelines are considered to be suitable in general, and they do fit with existing knowledge about post-trauma assistance, future research may refine and improve them. The ultimate test of their validity of the guidelines as a whole would be their ability to reduce risk for the development of mental disorders when evaluated in a controlled trial.

All panellists were recruited from developed English-speaking countries, so it is not expected that the guidelines will necessarily be generalisable to other countries or to minority cultures within those countries.

These guidelines are not a comprehensive guide to providing support, as they address only actions which may be useful after a traumatic event. The trauma may have ongoing effects regardless of intervention or the person may show few long term effects and recover quickly. First aid givers may find it useful to use these guidelines in conjunction with the other guidelines in this series, including first aid for depression, first aid for suicidal thoughts and behaviours, and first aid for non-suicidal self-injury [19-25]. These other guidelines can be downloaded from the Mental Health First Aid website [32].

These guidelines may not be suitable for use with people of all cultures. Guidelines have been developed for assisting Australian Aboriginal people who have experienced trauma and loss [33].

## Conclusions

This process has proven that it is possible to develop guidelines on how members of the public can provide mental health first aid following traumatic events, which are acceptable to both professionals and people who have been affected by traumatic events. These guidelines fit with what is currently known about risk factors for PTSD and other psychological sequelae of traumatic events [1,5,6]. However, these guidelines should be considered to be provisional, and need to be subject to further research. Where the guidelines are used as the basis for first aid training, efforts need to be made to evaluate their impact on the first aiders' helping behaviours and on the recipients of the first aid, as far as this is possible. This will assist researchers to develop an evidence base for mental health first aid and post-trauma intervention initiatives.

## Competing interests

The authors declare that they have no competing interests.

## Authors' contributions

CMK and AFJ prepared the manuscript. AFJ and BAK developed the methodology. CMK did the literature searches and wrote the first draft of the questionnaire. All authors contributed to the development of later versions of the questionnaire. CMK wrote the attached guidelines. All authors reviewed and suggested improvements to the guidelines. All authors read and approved this final manuscript.

## Pre-publication history

The pre-publication history for this paper can be accessed here:

http://www.biomedcentral.com/1471-244X/10/49/prepub

## Supplementary Material

Additional file 1 **First aid guidelines for traumatic events: adult version (PDF)**. This file may be distributed freely, with the authorship and copyright details intact. Please do not alter the text or remove the authorship and copyright details. Click here for file

Additional file 2 **First aid guidelines for traumatic events: child version (PDF)**. This file may be distributed freely, with the authorship and copyright details intact. Please do not alter the text or remove the authorship and copyright details. Click here for file
