# Supplementary material for: Development of mental health first aid guidelines on how a member of the public can support a person affected by a traumatic event: a Delphi study
Source: BMC Psychiatry. 2010 Jun 21;10:49. doi: 10.1186/1471-244X-10-49 (PMC2904289; doi:10.1186/1471-244X-10-49)
Supplement: Additional file 1 — First aid guidelines for traumatic events: adult version (PDF). This file may be distributed freely, with the authorship and copyright details intact. Please do not alter the text or remove the authorship and copyright details. [file 1471-244X-10-49-S1.PDF]

MENTAL HEALTH FIRST AID

TRAUMATIC EVENTS

FIRST AID GUIDELINES FOR ASSISTING ADULTS

What are the first priorities for helping someone after a traumatic event?

A ‘traumatic event’ is any incident experienced by the person that is perceived to be traumatic. Common examples of traumas that affect individuals include accidents (such as traffic, car or physical accidents), assault (including physical or sexual assault, mugging or robbery, or family violence), and witnessing something terrible happen. Mass traumatic events include terrorist attacks, mass shootings, and severe weather events (hurricane, tsunami, forest and bush fire).

Please note there are separate guidelines for assisting children who have experienced traumatic events

Mental health first aid might not always occur immediately after the traumatic event.

For instance, there are other sorts of traumas that are not single discrete incidents:

- Common examples of recurring trauma include sexual, physical or emotional abuse, torture, and bullying in the schoolyard or workplace. In these cases, mental health first aid guidelines will be used when the first aider becomes aware of what has been happening.
- Sometimes the memories of a traumatic event suddenly or unexpectedly return, weeks, months or even years afterwards. Again, mental health first aid guidelines will be used when the first aider becomes aware of this.

It is important to know that people can differ a lot in how they react to traumatic events:

- One person may perceive an event as deeply traumatic, while another does not.
- Particular types of traumas may affect some individuals more than others.
- A history of trauma may make some people more susceptible to later traumatic events, while others become more resilient as a result.

The MHFA Training & Research Program
Orygen Youth Health Research Centre
Department of Psychiatry
The University of Melbourne
AUSTRALIA

www.mhfa.com.au

MENTAL HEALTH FIRST AID

TRAUMATIC EVENTS

FIRST AID GUIDELINES FOR ASSISTING ADULTS

What are the first priorities for helping someone after a traumatic event?

You need to ensure your own safety before offering help to anyone. Check for potential dangers, such as fire, weapons, debris, or other people who may become aggressive, before deciding to approach a person to offer your help.

If you are helping someone who you do not know, introduce yourself and explain what your role is. Find out the person's name and use it when talking to them. Remain calm, and do what you can to create a safe environment, by taking the person to a safer location or removing any immediate dangers.

If the person is injured, it is important that their injuries are attended to. If you are able to, offer the person first aid for their injuries, and seek medical assistance. If the person seems physically unhurt, you need to watch for signs that their physical or mental state is declining, and be prepared to seek emergency medical assistance for them. Be aware that a person may suddenly become disoriented, or an apparently uninjured person may have internal injuries that reveal themselves more slowly.

Try to determine what the person's immediate needs are for food, water, shelter or clothing. However, if there are professional helpers nearby (police, ambulance, or others) who are better able to meet those needs, don't take over their role.

If the person has been a victim of assault, you need to consider the possibility that forensic evidence may need to be collected (e.g. cheek swabs, evidence on clothing or skin). Work with the person in preserving such evidence, where possible. For example, they may want to change their clothes and shower, which may destroy forensic evidence. It may be helpful to put clothing in a bag for police to take as evidence and suggest to the person that they wait to shower until after a forensic exam. Although collecting evidence is important, you should not force the person to do anything that they don't want to do.

Do not make any promises you may not be able to keep. For example, don't tell someone that you will get them home soon, if this may not be the case.

What are the priorities if I am helping after a mass traumatic event?

Mass traumatic events are those that affect large numbers of people. They include severe environmental events (such as fires and floods), acts of war and terrorism, and mass shootings. In addition to the general principles outlined above, there are a number of things you need to do.

Find out what emergency help is available. If there are professional helpers at the scene, you should follow their directions.

Be aware of and responsive to the comfort and dignity of the person you are helping, e.g., by offering the person something to cover themselves with (such as a blanket) and asking bystanders or media to go away. Try not to appear rushed or impatient.

Give the person truthful information and admit that you lack information when this is the case. Tell the person about any available sources of information which are offered to survivors (for example, information sessions, fact sheets and phone numbers for information lines) as they become available. Do not try to give the person any information they do not want to hear, as this can be traumatic in itself.

How do I talk to someone who has just experienced a traumatic event?

When talking to a person who has experienced a traumatic event, it is more important to be genuinely caring than to say all the "right things". Show the person that you understand and care, and ask them how they would like to be helped. Speak clearly and avoid clinical and technical language, and communicate with the person as an equal, rather than as a superior or expert. If the person seems unable to understand what is said, you may need to repeat yourself several times. Be aware that providing support doesn't have to be complicated; it can involve small things like spending time with the person, having a cup of tea or coffee, chatting about day-to-day life or giving them a hug.

Behaviour such as withdrawal, irritability and bad temper may be a response to the trauma, so try not to take such behaviour personally. Try to be friendly, even if the person is being difficult.

The person may not be as distressed about what has happened as you might expect them to be, and this is fine. Don't tell the person how they should be feeling. Tell them that everyone deals with trauma at their own pace. Be aware that cultural differences may influence the way some people respond to a traumatic event; for example, in some cultures, expressing vulnerability or grief around strangers is not considered appropriate.

Should we talk about what happened? How can I support someone in doing so?

It is very important that you do not force the person to tell their story. Remember that you are not the person's therapist.

Only encourage the person to talk about their reactions if they feel ready and want to do so. If the person does want to talk, don't interrupt to share your own feelings, experiences or opinions. Be aware that the person may need to talk repetitively about the trauma, so you may need to be willing to listen on more than one occasion.

Avoid saying anything that might trivialise the person's feelings, such as "don't cry" or "calm down", or anything that might trivialise their experience, such as "you should just be glad you're alive."

Be aware that the person may experience survivors' guilt; the feeling that it is unfair that others died, or were injured, while they were not.

How can I help the person to cope over the next few weeks or months?

If you are helping someone you know after a traumatic event, you can help them to cope with their reactions over the next few weeks or months. You may be helping a family member, perhaps a spouse, sibling or parent who you are living with. If you are helping someone you don't know, unless you are responsible for them in some professional capacity, it is not expected that you will have further contact with them.

Encourage the person to tell others when they need or want something, rather than assume others will know what they want. Also encourage them to identify sources of support, including loved ones and friends, but remember that it is important to respect the person's need to be alone at times.

TRAUMATIC EVENTS

FIRST AID GUIDELINES FOR ASSISTING ADULTS

Encourage the person to take care of themselves; to get plenty of rest if they feel tired, to do things that feel good to them (e.g., take baths, read, exercise, watch television), and to think about any coping strategies they have successfully used in the past and use them again. Encourage them to spend time somewhere they feel safe and comfortable.

Be aware that the person may suddenly or unexpectedly remember details of the event, and may or may not wish to discuss these details. If this happens, the general principles outlined above can help you to assist the person.

Discourage the person from using negative coping strategies such as working too hard, using alcohol and other drugs, or engaging in self-destructive behaviour.

When should the person seek professional help?

Not everyone will need professional help to recover from a traumatic event. If the person wants to seek help, you should support them to do so. Be aware of the sorts of professional help that are available locally, and if the person does not like the first professional they speak to, you should tell them that it is okay to try a different one. If the person hasn't indicated that they want professional help, the following guidelines can help you to determine whether help is needed.

If at any time the person becomes suicidal, you should seek professional help. The companion guidelines entitled First aid for suicidal thoughts and feelings may be useful in helping you to do this. Also, if at any time the person abuses alcohol or other drugs to deal with the trauma, you should encourage them to seek professional help.

After 4 weeks, some return to normal functioning is expected. You should encourage the person to seek professional help if, for 4 weeks or more, after the trauma:

- They still feel very upset or fearful.
- They are unable to escape intense, ongoing distressing feelings.
- Their important relationships are suffering as a result of the trauma (e.g., if they withdraw from their family or friends).
- They feel jumpy or have nightmares because of or about the trauma.
- They can't stop thinking about the trauma.
- They are unable to enjoy life at all as a result of the trauma
- Their post-trauma symptoms are interfering with their usual activities.

Purpose of these Guidelines

These guidelines are designed to help members of the public to provide first aid to someone who has experienced a traumatic event. The role of the first aider is to assist the person until appropriate professional help is received or the crisis resolves

Development of these Guidelines

The following guidelines are based on the expert opinions of a panel of mental health consumers, carers and clinicians from Australia, New Zealand, the UK, the USA and Canada about how to help someone who has experienced a traumatic event. Details of the methodology can be found in: (…)

How to use these Guidelines

These guidelines are a general set of recommendations about how you can help someone who has experienced a traumatic event. Each individual is unique and it is important to tailor your support to that person's needs. These recommendations therefore may not be appropriate for every person who has experienced a traumatic event.

Also, the guidelines are designed to be suitable for providing first aid in developed English-speaking countries. They may not be suitable for other cultural groups or for countries with different health systems.

Although these guidelines are copyright, they can be freely reproduced for non-profit purposes provided the source is acknowledged.

Please cite these guidelines as follows:
Mental Health First Aid Training and Research Program. *Traumatic events: first aid guidelines for assisting adults.* Melbourne: Orygen Youth Health Research Centre, University of Melbourne; 2008.

Enquiries should be sent to:

Professor Tony Jorm, Orygen Youth Health Research Centre
Locked Bag 10, Parkville VIC 3052 Australia
email: ajorm@unimelb.edu.au

8187_08_ORYGENKJ
