# Supplementary material for: Development of mental health first aid guidelines on how a member of the public can support a person affected by a traumatic event: a Delphi study
Source: BMC Psychiatry. 2010 Jun 21;10:49. doi: 10.1186/1471-244X-10-49 (PMC2904289; doi:10.1186/1471-244X-10-49)
Supplement: Additional file 2 — First aid guidelines for traumatic events: child version (PDF). This file may be distributed freely, with the authorship and copyright details intact. Please do not alter the text or remove the authorship and copyright details. [file 1471-244X-10-49-S2.PDF]

TRAUMATIC EVENTS

FIRST AID GUIDELINES FOR ASSISTING CHILDREN

MENTAL HEALTH FIRST AID

What is a traumatic event?

A 'traumatic event' is any incident experienced by the person that is perceived to be traumatic. Common examples of traumas that affect individuals include accidents (such as traffic, car or physical accidents), assault (including physical or sexual assault, mugging or robbery, or family violence), and witnessing something terrible happen. Mass traumatic events include terrorist attacks, mass shootings, and severe weather events (hurricane, tsunami, forest and bush fire).

If mental heath first aid is being provided by a parent, the parental role takes precedence over the first aid role.

Please note there are separate guidelines for assisting adults who have experienced traumatic events

Mental health first aid might not always occur immediately after the traumatic event.

For instance, there are other sorts of traumas that are not single discrete incidents:

- Common examples of recurring trauma include sexual, physical or emotional abuse, torture, and bullying in the schoolyard or workplace. In these cases, mental health first aid guidelines will be used when the first aider becomes aware of what has been happening.
- Sometimes the memories of a traumatic event suddenly or unexpectedly return, weeks, months or even years afterwards. Again, mental health first aid guidelines will be used when the first aider becomes aware of this.

It is important to know that people can differ a lot in how they react to traumatic events:

- One person may perceive an event as deeply traumatic, while another does not.
- Particular types of traumas may affect some individuals more than others.
- A history of trauma may make some people more susceptible to later traumatic events, while others become more resilient as a result.

The MHFA Training & Research Program
Orygen Youth Health Research Centre
Department of Psychiatry
The University of Melbourne
AUSTRALIA

www.mhfa.com.au

MENTAL HEALTH FIRST AID

TRAUMATIC EVENTS

FIRST AID GUIDELINES FOR ASSISTING CHILDREN

What are the first priorities for helping a child after a traumatic event?

You need to ensure your own safety before offering help to anyone. Determine whether it is safe to approach the child. Before deciding to approach a child to offer your help, check for potential dangers (for example, from fire, weapons, or debris), including any person who may become aggressive.

If you are helping a child who you do not know, introduce yourself and explain that you are there to help. Find out the child's name and use it when talking to them. Remain calm. Do what you can to protect the child (whether by taking them to a safer location or removing any immediate dangers). Reassure the child that they won't be left alone, so far as this is possible, and ensure that you, or another adult (such as a professional helper), are available to take care of the child. If you have to leave the child alone for a few minutes to attend to others, reassure the child that you will back soon. However, try not to behave towards the child in such a way that they feel they are still in danger.

If the child is injured, it is important that their injuries are attended to. If you are able to, give the child first aid for their injuries, and seek medical assistance. If the child seems physically unhurt, you need to watch for signs that their physical or mental state is declining, and be prepared to seek emergency medical assistance for them. Be aware that a child may suddenly become disoriented, or an apparently uninjured child may have internal injuries that reveal themselves more slowly.

Try to determine what the child's immediate needs are for food, water, shelter or clothing. However, if there are professional helpers nearby (police, ambulance, or others) who are better able to meet those needs, don't take over their role.

Don't make any promises you may not be able to keep. For example, don't promise the child that you will get them home soon, when this may not be the case.

What are the priorities if I am helping after a mass traumatic event?

Mass traumatic events are those which affect large numbers of people. They include severe environmental events (such as fires and floods), acts of war and terrorism, and mass shootings. In addition to the more general guidelines in the previous section, there are a number of things you need to do.

Try to keep the child together with any loved ones and carers who are present. If they are not present, or have been separated from the child in the course of the event, ensure that the child is re-connected with them as soon as possible.

Ask the child what would make them feel better or safer. Direct the child away from traumatic sights and sounds (including media images), people who are injured, and very distressed people (e.g., anyone who is screaming, agitated or aggressive). Ask bystanders and the media to stay away from the child.

How do I talk to a child who has experienced a traumatic event?

This section of the guidelines may be used to help you support a child after they have experienced a traumatic event. If you know the child, then you can use these guidelines to offer the child ongoing support. If you don't know the child, then you can use these as a guide for talking to the child at anytime that you come into contact with them following their traumatic experience, e.g. at the scene of a trauma, or later on, at home, in a classroom, or elsewhere.

Remember, when talking to a child who has experienced a traumatic event, that it is more important to be genuinely caring than to say all the "right things". Show the child that you understand and care, be patient, and tell the child you will do your best to keep them safe.

Talk to the child using age-appropriate language and explanations. Allow the child to ask questions and answer them as truthfully as possible. Be patient if the child asks the same question many times, and try to be consistent with answers and information. If you can't answer a question, admit to the child that you don't know the answer.

If the child knows accurate, upsetting details, don't deny these. When someone has died, it can be tempting to soften this news by telling a child that the person has "gone to sleep", but this is best avoided, as it may result in the child becoming fearful of sleep.

A child may stop talking altogether after a trauma, and if this happens, you should not try to force or coerce the child to speak. Equally, you should never coerce a child to talk about their feelings or memories of the trauma before they are ready to do so.

If the child wants to talk about their feelings, you should allow them to. Some children prefer to express their feelings through writing, drawing, or playing with toys.

Never tell the child how they should or shouldn't be feeling. Don't tell the child to be brave, or not to cry, and don't make judgements about their feelings. Don't get angry if the child expresses strong emotions; instead tell them it is okay to feel upset when something bad or scary happens

A child has told me that they are being abused. What should I do?

Remain calm and reassure the child that they have done the right thing by telling you, and that what happened was not their fault. Tell the child that you believe them.

You need to know the local laws or regulations about reporting suspected child abuse and follow these. Contact the appropriate authorities and work with them to ensure the child's safety. Do not confront the perpetrator

I am a parent/guardian and the child I am helping lives with me. How should I behave at home?

Try to keep your behaviour as predictable as possible, and tell the child that you (and their other loved ones) love and support them. Encourage the child to do things they enjoy, such as playing with toys or reading books. You can help the child to feel in control by letting them make some decisions (e.g. about meals, or what to wear).

Dealing with temper tantrums and avoidance behaviours

Be aware that the child may avoid things that remind them of the trauma (such as specific places, driving in the car, certain people, or separation from their parents or guardians). Try to figure out what triggers sudden fearfulness or regression in the child. If the child has temper tantrums or becomes fearful, crying and clingy in order to avoid something that reminds them of the trauma, ask them what they are afraid of. Don't get angry or call the child 'babyish' if they appear to regress, for example by bedwetting, misbehaving, or sucking their thumb.

If the child avoids things which remind them of the trauma, but does not appear very distressed, ask what they are afraid of and assure them that they are safe.

The symptoms associated with trauma may suddenly or unexpectedly appear months or years after the event. If this occurs, professional help may need to be sought.

TRAUMATIC EVENTS

FIRST AID GUIDELINES FOR ASSISTING CHILDREN

MENTAL HEALTH FIRST AID

Should the child receive professional help?

Not all children will need professional help to recover from a traumatic event.
The following guidelines can help you to determine whether help is needed.

If at any time the child becomes suicidal, you should seek immediate professional help.

You should seek professional help for the child if, for 2 weeks or more after the trauma:

- The child is unable to enjoy life at all.
- The child displays sudden severe or delayed reactions to trauma.
- The child is unable to escape intense ongoing distressing feelings.
- The child's post-trauma symptoms are interfering with their usual activities.
- The child's important relationships are suffering (e.g. if they withdraw from their carers or friends).

You should seek professional help for the child if, for 4 weeks or more after the trauma:

- The child has temper tantrums or becomes fearful, crying and clingy in order to avoid something which reminds them of what happened.
- The child still feels very upset or fearful.
- The child acts very differently compared to before the trauma.
- The child feels jumpy or has nightmares because of or about the trauma.
- The child can't stop thinking about the trauma.

You should be aware of the types of professional help which are available locally for children. Clinical child psychologists, psychiatrists, pediatricians and family doctors can all be helpful. If you are not the child's parent or guardian, do not seek professional help for them unless it is an emergency; instead assist the child's parent or guardian to seek professional help for them.

Purpose of these Guidelines

These guidelines are designed to help members of the public to provide first aid to a child who has experienced a traumatic event. The role of the first aider is to assist the person until appropriate professional help is received or the crisis resolves.

Development of these Guidelines

The following guidelines are based on the expert opinions of a panel of mental health consumers, carers and clinicians from Australia, New Zealand, the UK, the USA and Canada about how to help someone who has experienced a traumatic event. Details of the methodology can be found in: (…)

How to use these Guidelines

These guidelines are a general set of recommendations about how you can help a child who has experienced a traumatic event. Each child is unique and it is important to tailor your support to that child's needs. These recommendations therefore may not be appropriate for every child who has experienced a traumatic event.

Also, the guidelines are designed to be suitable for providing first aid in developed English-speaking countries. They may not be suitable for other cultural groups or for countries with different health systems.

Although these guidelines are copyright, they can be freely reproduced for non-profit purposes provided the source is acknowledged.

Please cite these guidelines as follows:
Mental Health First Aid Training and Research Program. *Traumatic events: first aid guidelines for assisting children.* Melbourne: Orygen Youth Health Research Centre, University of Melbourne; 2008.

Enquiries should be sent to:

Professor Tony Jorm, Orygen Youth Health Research Centre
Locked Bag 10, Parkville VIC 3052 Australia
email: ajorm@unimelb.edu.au

8186_08_ORYGENKJ
